# Diffuse Calcifications of the Spleen in a Woman with Systemic Lupus Erythematosus

**DOI:** 10.1155/2015/414102

**Published:** 2015-08-17

**Authors:** Aristeides G. Vaiopoulos, Meletios A. Kanakis, Kyriaki Katsouri, Stavroula Kyriazi, George A. Vaiopoulos, Phaedon Kaklamanis

**Affiliations:** ^1^Department of Experimental Physiology, School of Medicine, University of Athens, 75 Mikras Asias Street, 11527 Athens, Greece; ^2^Medical Center of Athens, 5-7 Distomou Street, 15125 Marousi, Greece

## Abstract

Systemic lupus erythematosus (SLE) is a multisystemic autoimmune disease, which affects a wide variety of organs including the spleen. Splenic involvement in SLE includes conditions such as splenomegaly, hyposplenism, infarction, and spontaneous rupture. However, only a few cases of splenic calcifications in patients with SLE have been reported. Herein, we present a case of a 24-year-old female diagnosed with SLE, in which we found diffuse splenic calcifications. The unique pattern of splenic calcifications in SLE contributes to the differential diagnosis from other conditions such as infections and other connective tissue diseases, which also cause calcifications in the spleen.

## 1. **Introduction**

Systemic lupus erythematosus (SLE) affects various organs including the spleen. Splenomegaly, hyposplenism, infarction, spontaneous rupture, functional asplenia, and periarterial thickening in an “onion skin” pattern have been recognized in SLE patients [1–4]. Splenic calcifications have been reported in connective tissue disorders such as rheumatoid arthritis, systemic sclerosis [5], infections, sickle cell disease, splenic haemangiomas, and cysts, and in B-cell lymphoma [6]. However, SLE has not been widely recognized as a cause of splenic calcifications and few patients with this abnormality have been reported [1, 7, 8]. This abnormality has been recently associated with SLE [1]. Herein, we report a case of a woman with SLE who was found to have diffuse splenic calcifications.

## 2. **Case Presentation**

A 24-year-old female employed in a Greek bank was diagnosed with SLE at the age of 18. The patient presented with arthralgia, diffuse rash, and vasculitis. The diagnosis was confirmed with positive serology. Laboratory investigation showed antinuclear antibodies (>1: 640) with diffuse pattern, anti-dsDNA (>94 U/mL, *N*: 0–7 U/mL), anti-Ro (+), immunoglobulin IgG (1840 mg/dL, *N*: 690–1618 mg/dL), and *γ*-globulin (23.9%, *N*: 10.5–19.5%) on electrophoresis. The patient was treated initially with corticosteroids (prezolon) 40 mg/d with tapering according to clinical manifestations and hydroxychloroquine 400 mg/d.

During the following years, autoantibodies were always positive, although varying in titles. Blood urea nitrogen (43.0 mg/dL, *N*: 10–50 mg/dL) and serum creatinine (0.67 mg/dL, *N*: 0.6–1.4 mg/dL) were in a normal range.

Urine analysis was negative for the presence of protein and casts during the years of observation until the last two years. In the last two years, CH50 (20.3 u/mL, *N*: 23.0–46.0 u/mL), C3 (72.0 mg/L, *N*: 90–180 mg/L), and C4 (9.0 mg/L, *N*: 10–40 mg/L) were low. A 24-hour urine specimen in February 2013 showed proteinuria, ranging from 0.300 gr/24 h to 2.0 gr/24 h. IgM cardiolipin was increased (38.0 MPL, *N* < 12.5 MPL). ESR was (45 mm/1 h). Following the previous laboratory results particularly the proteinuria and the clinical findings (arthralgia, digital vasculitis, and discoid-like lesions exacerbated), a transcutaneous needle biopsy of the kidney was performed. On examination, there were no findings from the chest and abdominal organs. Blood pressure was 130/75 mm Hg and pulses were 60–65/min. Histological examination revealed the presence of focal lupus nephritis, with an active disease index 10 (*N*: 0–24) and chronicity index 1 (*N*: 0–12). Classification according to ISN/RPS was defined as Class III (A/C).

**Figure 1:**
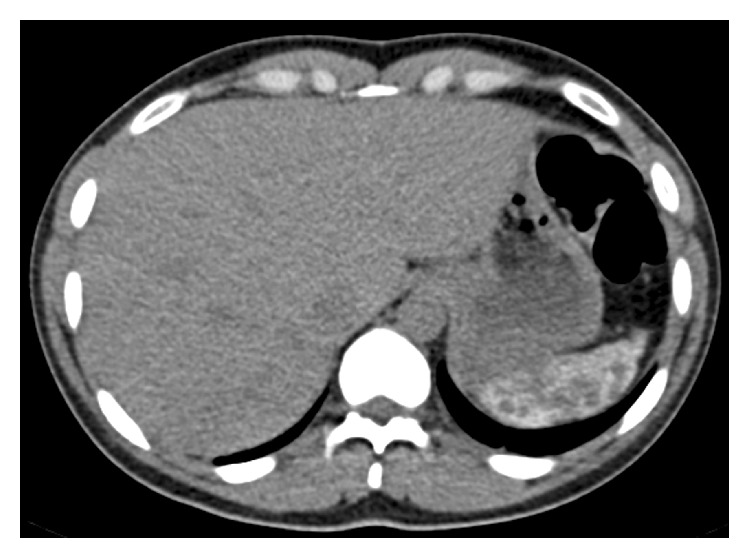
Computed tomography scan section, depicting diffuse calcifications of the spleen.

During abdominal ultrasound examination, calcified foci in the splenic area were incidentally noted. A subsequent, abdominal computed tomography (CT) scan was performed, which showed diffuse calcifications of the spleen (Figure 1).

Mantoux test was negative and the chest CT scan was also normal. Screening test for sickle cell hemoglobin was negative. Infections, other connective tissue diseases, and lymphoma were excluded from the history, clinical examination, and laboratory findings.

Recently, discoid-like lesions mainly on the patient's face and vasculitis of the fingers have been exacerbated. The patient continued receiving corticosteroids (prezolon 30–40 mg/d), hydroxychloroquine (400 mg/d) and we added mycophenolic acid (2 gr daily).

## 3. **Discussion**

Systemic lupus erythematosus (SLE) is a multisystemic autoimmune disease affecting all organs, including the spleen. Rupture, splenomegaly, infarction, infections, and atrophy of the spleen have been recognized in patients with SLE.

Splenic calcifications have been reported in various diseases except for SLE such as rheumatoid arthritis, systemic sclerosis, amyloidosis, sickle cell anemia, anthrasilicosis, lymphoma, overlap syndrome, infections, trauma, and celiac disease [5, 6, 9, 10].

In our case, the known causes of splenic calcifications such as infections (including tuberculosis), sickle cell disease, lymphoma, and environmental causes were excluded from the history, clinical examination, and laboratory findings, as these should be considered in every such case. Segmental splenic infarction is associated with anti-cardiolipin antibodies and our patient had also positive anti-cardiolipin antibodies [11]. However, the calcification pattern is different in infection and other non-SLE abnormalities [1]. Tieng and coworkers [1] have proposed that splenic calcifications in SLE patients follow a unique pattern. Diffuse splenic calcifications which are predominantly discrete, small, rounded, and larger than the punctuate calcifications seen with granulomatous infections seem to be specific for SLE [1, 5]. Moreover, splenic calcifications in other connective tissue disorders seem to be isolated, smaller, less dense, and closer to the capsular region than those found in SLE [5]. Clinicians should be aware of these different patterns of splenic calcifications.

Whether splenic calcification can predispose to hyposplenism remains to be determined [1]. It is possible that splenic microcalcifications could be the late-end consequence of an immune-mediated inflammation of the arterial vessels probably linked to repeated flares of SLE [8].

In order to determine the significance and the aetiopathogenesis of the diffuse calcifications of the spleen in SLE patients, further studies are needed.
